# Antifungal Activity of an Abundant Thaumatin-Like Protein from Banana against *Penicillium expansum*, and Its Possible Mechanisms of Action

**DOI:** 10.3390/molecules23061442

**Published:** 2018-06-14

**Authors:** Wenxiao Jiao, Xiangxin Li, Handong Zhao, Jiankang Cao, Weibo Jiang

**Affiliations:** College of Food Science and Nutritional Engineering, China Agricultural University, 17 Qinghuadonglu Road, Beijing 100083, China; DC_COCOA@OUTLOOK.COM (W.J.); lixiangxin2016@163.com (X.L.); h_d_zhao@sina.com (H.Z.); cjk@cau.edu.cn (J.C.)

**Keywords:** banana, thaumatin-like protein, antifungal activity, *Penicillium expansum*, mechanisms of action

## Abstract

Thaumatin-like protein from banana (designated BanTLP) has been purified by employing a simple protocol consisting of diethylaminoethyl Sephadex (DEAE–Sephadex) chromatography, gel filtration on Sephadex G50, and reversed-phase chromatography. The purified protein was identified by MALDI-TOF mass spectrometry, with an estimated molecular weight of 22.1 kDa. BanTLP effectively inhibited in vitro spore germination of *Penicillium expansum*, one of the main postharvest pathogens in fruits. This study further investigated the antifungal properties and underlying mechanisms of BanTLP against *P. expansum*. Results demonstrated that BanTLP exhibited antifungal activity in a wide pH range (4.0–10.0) at 20–50 °C. Propidium iodide (PI) influx and potassium release confirmed that BanTLP induced membrane disruption of the test pathogen, increasing the membrane permeability and disintegration of the cell. This led to cell death, as evidenced by the assays of thiobarbituric acid-reactive species (TBARS) content, the production of reactive oxygen species (ROS), and 1,6-diphenyl-1,3,5-hexatriene (DPH) fluorescence integrity. Ultrastructural alterations in *P. expansum* conidia after BanTLP treatment revealed severe damage to the cell wall. These results suggest that BanTLP purified from banana exerts antifungal activity against *P. expansum* by inducing plasma membrane disturbance and cell wall disorganization.

## 1. Introduction

Banana (*Musa acuminate*) is considered to be one of the most important and popular fruits in the human diet due to its pleasant taste and its high nutritional value, in addition to its anti-hypertension, anti-diabetic, anti-ulcerogenic, anti-cancer, anti-proliferative, and antioxidant functions [1]. Research has shown that the pulp of ripe banana contains a great quantity of thaumatin-like protein (TLP) [2]. 

TLP, which belongs to pathogenesis-related (PR) protein family 5 (PR5 protein), shares an amino acid sequence with thaumatin (a sweet-tasting protein originally found in the arils of the ripe West African rain forest shrub *Thaumatococcusdanielli*), and is structurally similar [3]. TLPs are the products of a large, highly complex gene family containing fungal, plant, and animal TLPs. TLPs appear to possess biological functions such as antifungal, glucanase, and xylanase inhibition activities, and are considered to be involved in host defense, stress tolerance, and cell signaling [4,5,6]. Recently, many transgenic plants overexpressing TLPs have been developed for enhancing host resistance against pathogens, indicating that TLPs hold a great prospective for the molecular breeding of resistant plants [3].

TLPs isolated from banana, peach, and kiwifruit have been proven to be potent antifungal proteins [7,8,9]. However, this activity is reportedly lacking in other TLPs in plants [2,4]. An early study by Koiwa explained that the efficacy of TLPs depends on the combination of fungal species and TLP isoforms [10]. During the last two decades, much attention has been drawn to revealing the underlying mechanism of antifungal activity of TLPs, but to date it is still poorly understood. It has been reported previously that there is a direct relationship between the antifungal activities of TLPs and their ability to bind to several water-insoluble β-1,3-glucans, cell wall components of pathogenic fungi [4]. A molecular docking assay conducted by Liu further confirmed that Asp and Glu residues found in the cleft of soybean hulls TLP interacted with linear β-1,3-glucans through an inverting or retaining mechanism of glucan hydrolysis [11]. However, these previous studies focused on the analysis of the structure features underlying these antifungal activities, with emphasis on the molecular mechanism. Few studies have investigated the physiological and ultrastructural changes in fungi exposed to TLPs. This work will lead to new insights into the mechanism of action of the antifungal protein against fungi. 

*P. expansum* is a devastating pathogen in fruit, leading to considerable economical losses during the postharvest storage and handling processes [12]. It has been reported that TLP purified from basrai banana can be used as a preservative in the food processing industry due to its inhibitory effect on the growth of *Aspergillus phoenicis* and *Aspergillus flavus* on white bread [7]. Another example for application of TLP would be the protection of rye bread against *Penicillium requeforti*, a major contaminating strain in the food processing industry [13]. To our knowledge, this work investigates for the first time the effect of BanTLP on the postharvest pathogen fungi (*P. expansum*) in order to promote the application of TLP for direct crop protection and gene engineering.

This study aimed to: (1) validate the antifungal activity of BanTLP isolated and purified from the banana fruit against *P. expansum*; and (2) observe the physiological and ultrastructural changes in *P. expansum* exposed to BanTLP and elucidate its mechanisms of action.

## 2. Results and Discussion

### 2.1. Purification and Identification of Thaumatin-Like Protein from Banana (Ban TLP)

**Figure 1:**
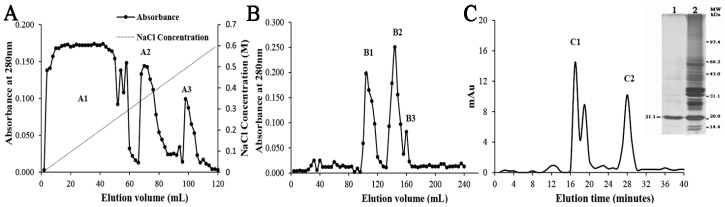
Purification of BanTLP. (**A**) Ion-exchange chromatography of the fraction of banana fruit extract (crude banana extract) with DEAE–Sephadex chromatography A50. The target protein was only observed in fraction A1. (**B**) Gel filtration of fraction A1 by fast protein liquid chromatography on a Sephadex G50 column. The target protein was observed in fractions B2 and B3. (**C**) The active fractions from gel filtration were pooled and used in a reverse-phase Supelco C5 column. The target protein was observed in fraction C2. (Insert) SDS-PAGE profiles of purified antifungal protein from banana fruit visualized using a silver-staining technique. Lane 1: the target protein; Lane 2: crude banana extract. Numbers on the right refer to molecular mass markers. MW represents the molecular weight marker.

BanTLP has been purified using a three-step chromatographic purification procedure consisting of anion-exchange chromatography, gel filtration, and reversed-phase chromatography, whereby the elutes were monitored by determining the absorbance at 280 nm. A crude extract of banana fruit was applied to a DEAE–Sephadex A50 column eluted with a gradient of NaCl in the buffer, and three major fractions (A1, A2, and A3) were obtained (Figure 1A). A broad absorbance peak (A1) with immunocompetence against the BanTLP antibody was further purified by Sephadex G50 on the basis of molecular mass. After gel filtration, peak B1 was devoid of immunocompetence, and peaks B2 and B3 with immunocompetence were obtained (Figure 1B). The active fractions were pooled and subjected to reversed-phase chromatography on a C5 column, and peak C2 with immunocompetence was obtained (Figure 1C). The purity of BanTLP was confirmed by SDS-PAGE, which revealed that the purified protein consisted exclusively of a single polypeptide (Figure 1C, insert). The purified protein was identified as thaumatin-like protein with a molecular weight of 22.1 kDa by mass spectrometry analysis (see Supplementary Materials Figure S1), and the Mascot score was 414 (greater than 77 is significant, *p* < 0.05). A yield of 58 mg of pure BanTLP was obtained from 1.5 kg of banana pulp (Table 1). This combination of different chromatograph techniques forms an efficient isolation method for bioactive compounds from plants, and is used extensively in protein purification. This work proposed a three-step chromatographic purification procedure to purify an abundant protein (BanTLP) from banana, which had similar chromatographic behaviors to thaumatin proteins purified from plants in previous studies [2,9].

### 2.2. Antifungal Properties of BanTLP against P. expansum

**Figure 2:**
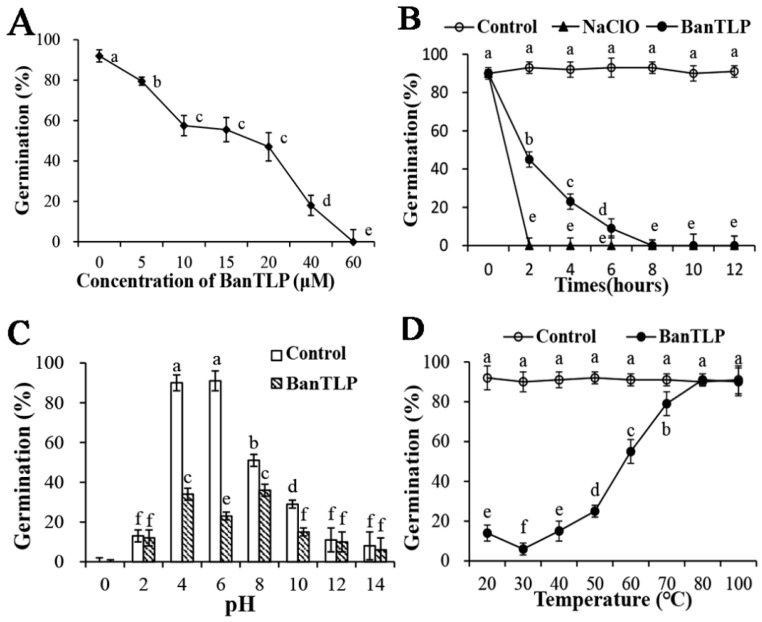
The effects of concentration (**A**), contact time (**B**), pH (**C**), and temperature (**D**) on the spore germination of *Penicillium expansum* exposed to BanTLP in potato dextrose broth (PDB). The germination rate of *P. expansum* was determined after 12 h of incubation at 28 °C. Each value is the mean of three replicates for two independent experiments. Vertical bars represent standard deviations of means. Those data marked with different letters are significantly different (*p* < 0.05) according to Duncan’s multiple range test.

TLPs are classified as forming part of the pathogenesis-related (PR) protein family 5 because they can be induced by biotic and abiotic stresses such as microbial infection, plant hormones, osmotic stress, and wounding. In previous study, TLPs were isolated and purified from various fruits. The antifungal protein isolated from kiwifruit showed a remarkable inhibitory effect on *Botrytis cinerea* and some suppressive effects on *Mycosphaerella arachidicola* and *Coprinus comatus* [9]. In another study, antifungal activity was observed in *Verticillium albo-atrum*, which exhibited sensitivity to BanTLP purified from banana [4]. However, so far, there are few detailed reports on the antifungal properties of TLP. Hence, this work examined its effects and the mechanisms involved. In the present study with BanTLP at 20 μM, the spore germination of *P. expansum* was reduced to about half that of the control, with a germination rate of 47%. BanTLP at 60 μM was able to completely inhibit the spore germination of *P. expansum* (Figure 2A) at 28 °C. Our result can be related to early reports described by Yasmin [7], who found that TLP isolated from banana inhibited the growth of *Fusarium oxysporum*, *Aspergillus niger*, *Aspergillus fumigatus*, and *Trichoderma viride*, with IC_50_ values of 9.7 μM, 11.83 μM, 4.61 μM, and 21.43 μM, respectively.

Sodium hypochlorite is an efficient and quick antifungal agent for commercial use. We investigated the effect of hypochlorite (0.05% of active chlorine) and BanTLP (60 μM) on the germination of *P. expansum* conidia after 12 h of incubation at 28 °C, using hypochlorite as a positive control. The results showed that BanTLP was effective at reducing the spore germination by up to 100% after 8 h of exposure treatment of conidia, but the effect was less than that of hypochlorite, which repressed 100% the spore germination after just 2 h of treatment (Figure 2B).

The biological functions of TLP can be directly influenced by pH and temperature, although its structure is considered to be stabilized due to the high number of disulfide bonds [14,15]. Results from this study indicated that the germination of *P. expansum* conidia was strongly inhibited both in control and treatment groups when pH values of the cultures media were lower than 2 or higher than 12, in agreement with the findings of Li (Figure 2C) [16]. After 12 h of exposure of the conidia, BanTLP significantly suppressed the germination of *P. expansum* conidia when pH values ranged from 4 to 10, with an optimal antifungal activity at pH 6. Additionally, a decrease in the antifungal activity was observed at pH 10, and completely loss of antifungal activity occurred at higher pH values. The thermal stability of BanTLP was examined and the antifungal activity was stable at temperatures ranging from 20 to 50 °C, with optimal antifungal activity at 30 °C (Figure 2D). A comparison of the observations in this work with those of Ho indicates that antifungal activity of TLP isolated from emperor banana is more stable in a wider pH range (pH 1 to 11) and up to 70 °C [17]. These differences may be ascribed to different fungal species and analysis methods. Similarly, Menu found that BanTLP isolated from banana exhibited endo-β(1,3)-glucanase activity in a pH-dependent manner, with optimal activity at pH 5 [4].

### 2.3. Effect of BanTLP on Membrane Permeability and Disturbance in P. expansum

**Figure 3:**
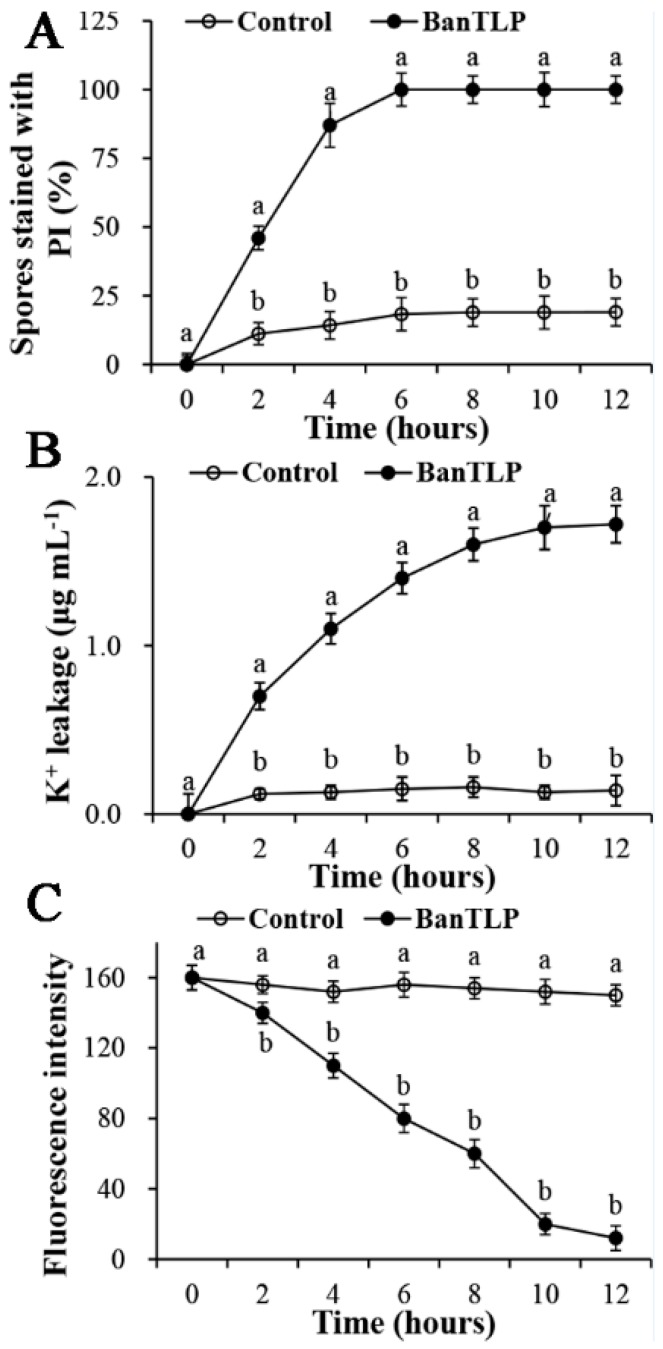
Effects of BanTLP at 60 μM on the plasma membrane of *P. expansum* conidia as the exposure times extended. (**A**) Spores stained with propidium iodide (PI); (**B**) the leakage of intracellular potassium ions; (**C**) 1,6-diphenyl-1,3,5-hexatriene (DPH) fluorescence intensity of *P. expansum.* Each value is mean of three replicates for two independent experiments. Vertical bars represent standard deviations of means. Those data marked with different letters are significantly different (*p* < 0.05) between control and treatment groups at the same time according to Duncan’s multiple range test.

The integrity of mycelia membrane of *P. expansum* was investigated after exposure to 60 μM BanTLP by monitoring the propidium iodide (PI) influx. PI is a DNA-staining fluorescent probe that can enter the cell if the plasma membrane of cell is disrupted, resulting in a nucleus stained with a red fluorescent color [18]. Results in Figure 3A indicate that fluorescent intensity increased in conidia of *P. expansum* exposed to BanTLP as the incubation times extended. After 4 h of incubation with BanTLP, the percentage of PI fluorescent-stained conidia was 87%, while that of the control was 14.2%. After 6 h of incubation, 100% of conidia of *P. expansum* were stained in BanTLP-treated group, while only 18.3% were stained in the control group. The result of PI influx indicated that BanTLP is able to induce the plasma membrane disruption. The corresponding fluorescence images of PI influx is displayed in Supplementary Materials Figure S2.

The potassium ion release assay was conducted to estimate the permeability of the plasma membrane. Compelling evidence from early studies indicated that the plasma membrane of toxin-damaged cells became permeable for monovalent ions, with subsequent potassium ions release from the cell [19]. K^+^ homeostasis may play a critical role in the maintenance of the membrane potential. Results in this study showed that the concentration of extracellular K^+^ significantly increased in the initial 10 h of incubation with BanTLP. In contrast, for the control treatment, the concentration of extracellular K^+^ was maintained at a low level throughout the experiment, with little K^+^ release (Figure 3B).

A 1,6-diphenyl-1,3,5-hexatriene (DPH) fluorescence assay was performed to estimate the plasma membrane perturbation in terms of lipid bilayer. The DPH probe can be buried completely in the bilayer core with no effect on the membrane structure. Any disorder in the membrane lipid bilayer was a result of DPH parting from the membrane, with a subsequent decrease in the DPH fluorescent polarization [20]. The results in Figure 3C show that the DPH fluorescent intensity descended remarkably in conidia of *P. expansum* exposed to BanTLP as the incubation times extended. After 6 h of incubation, the fluorescent intensity of conidia exposed to BanTLP was 48.7% lower than that of the control (*p* < 0.05). The result indicated that BanTLP treatment perturbed the membrane lipid bilayer of the test fungus, resulting in DPH detachment and a decrease in fluorescent intensity. However, for the control treatment, the fluorescent intensity was maintained as the incubation times increased, indicating the membrane lipid bilayer was stable and well-organized.

Previous studies investigated a number of antifungal agents (such as isoquercitrin, monocaprin, citral, and so on) that directly or indirectly attack the plasma membranes of pathogenic fungi, providing much insight into membrane permeabilization [21,22]. Through PI influx, potassium ion leakage, and DPH assays, this study confirmed the hypothesis that BanTLP caused the damage of cell by inducing membrane permeabilization and disturbances. Some studies on the structure of TLPs isolated from plants supported the idea that the membrane-permeabilizing activity of TLP might be ascribed to its acidic cleft [10]. It has been demonstrated that when positively-charged TLP isolated from soy hulls and the negatively-charged microbial membranes are combined superficially and electrostatically, the TLP partially enters the hydrophobic part of cytoplasmic membranes [11]. Therefore, much research is needed to investigate the deeper mechanisms of BanTLP acting as a membrane-permeabilizing protein against *P. expansum*.

### 2.4. Oxidative Stress in P. expansum Induced by BanTLP

**Figure 4:**
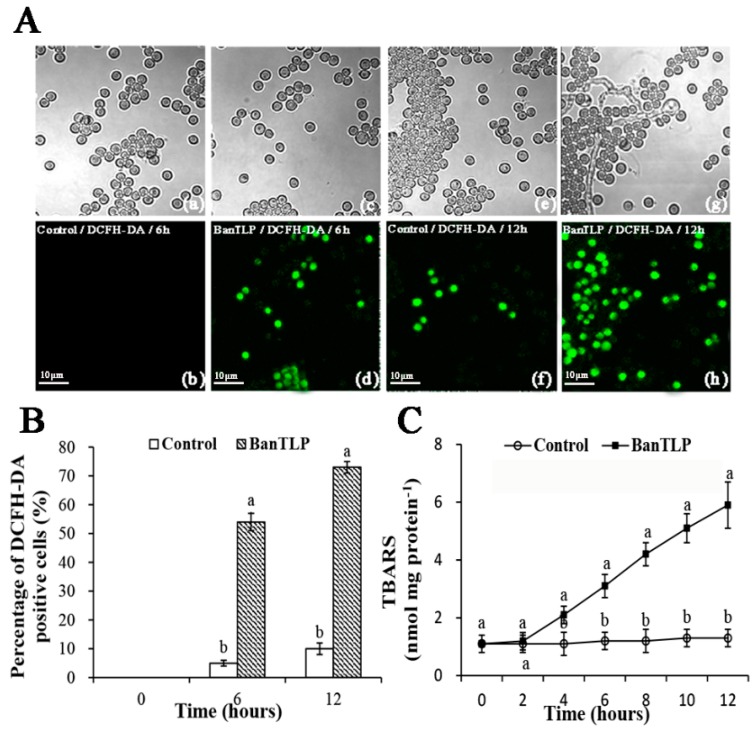
Effects of BanTLP at 60 μM on oxidative stress of *P. expansum.* Figures panels show images of (**A**) the generation of intracellular reactive oxygen species (ROS), (**B**) the percentage of 2′,7′-dichlorofluorescein diacetate (DCFH-DA) positive cells, and (**C**) thiobarbituric acid-reactive species (TBARS) contents. The DCFH-DA fluorescent probe was visualized with the aid of fluorescence microscopy in *P. expansum* conidia treated with distilled water for 6 h (**a**,**b**) and 12 h (**e**,**f**) or treated with BanTLP for 6 h (**c**,**d**) and 12 h (**g**,**h**). Upper panels display the bright field images, while lower panels display the corresponding fluorescence images for the same field. The scale bar represents 10 μm. Each value is mean of three replicates for two independent experiments. Vertical bars represent standard deviations of means. Those data marked with different letters are significantly different (*p* < 0.05) between control and treatment groups at the same time according to Duncan’s multiple range test.

Reactive oxygen species (ROS) and thiobarbituric acid-reactive species (TBARS) are considered oxidative stress biomarkers. The accumulation of ROS molecules, including superoxide, hydrogen peroxide, and hydroxyl radicals, can lead to detrimental stress responses in fungi, disrupting the cell physiological status and causing oxidative death [23]. ROS were determined using a 2′,7′-dichlorofluorescein diacetate (DCFH-DA) fluorescent probe, which has proven efficiency for characterizing the overall ROS generation in many biological materials. As shown in Figure 4A,B, after 6 h of incubation with BanTLP, the percentage of cells marked with intracellular ROS in *P. expansum* increased 9.8-fold compared to the control. Moreover, when the incubation time was increased up to 12 h, a 6.3-fold increase in the intracellular ROS generation was observed in the BanTLP treatment group compared to that of the control group (*p* < 0.05).

TBARS is the primary product of polyunsaturated fatty acids peroxidation, and can be used as an indicator of oxidative damage of the fungal plasma membrane. The variations in amounts of TBARS further confirmed the results from the ROS assay. When conidia of *P. expansum* were incubated with BanTLP at 28 °C for 6 h or 12 h, TBARS contents were 2.58- and 4.54-fold compared to that of the control, respectively (*p* < 0.05). Figure 4C shows that TBARS contents in conidia of *P. expansum* prepared in BanTLP continuously increased as the contact times increased, whereas that of the control showed no variation throughout the experiment. 

Similarly, the previous study demonstrated that osmotin, a tobacco PR-5 family protein, activated the RAS2/cAMP pathway and induced hyperproduction of intracellular ROS to promote programmed cell death of the yeast *Saccharomyces cerevisiae* [24]. Additionally, Hayea described a promising antifungal defensin, NaD1, with activity against *Candida albicans* because NaD1 induced ROS generation, leading to cell death as the result of oxidative damage [25]. Hence, BanTLP in this work promoted the accumulation of intracellular ROS, resulting in oxidative stress in *P. expansum*, which can be explained as another possible fungicidal action of BanTLP.

### 2.5. Effect of BanTLP on Cell Wall Integrity in P. expansum

**Figure 5:**
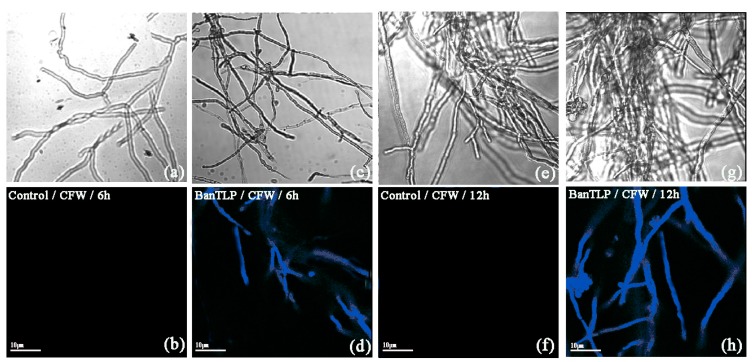
Effect of BanTLP at 60 μM on the cell wall integrity of *P. expansum* conidia. The Calcofluor White (CFW) fluorescent probe was visualized with the aid of fluorescence microscopy in *P. expansum* conidia treated with distilled water for 6 h (**a**,**b**) and 12 h (**e**,**f**) or treated with BanTLP for 6 h (**c**,**d**) and 12 h (**g**,**h**). Upper panels display the bright field images, while lower panels display the corresponding fluorescence images for the same field. The scale bar represents 10 μm.

The effect of BanTLP on *P. expansum* cell wall was evaluated using a Calcofluor White (CFW) fluorescent probe. CFW can monitor abnormal chitin alterations located in the cell walls of fungi through a specific combination with chitin [26]. In this study, after being treated with BanTLP at 60 μM for 6 h, a bright blue fluorescent signal was detected. When the incubation time was increased to 12 h, the bright blue fluorescence was enhanced in conidia cells in the treatment group. There was no apparent fluorescent signal in conidia cells in the control group throughout the experiment (Figure 5). The result from the present study indicated that BanTLP treatment severely damaged the cell wall of *P. expansum* conidia. Similarly, barley TLPs exerted their antifungal activity by binding to nascent β-1,3-glucans during fungal wall synthesis, preventing the growth of fungi [27]. The previous study confirmed that TLPs from plant could induce disruption of the fungal cell wall, leading to cell death. 

### 2.6. Morphological and Ultrastructural Changes of P. expansum Conidia

#### 2.6.1. Scanning Electron Microscope (SEM)

**Figure 6:**
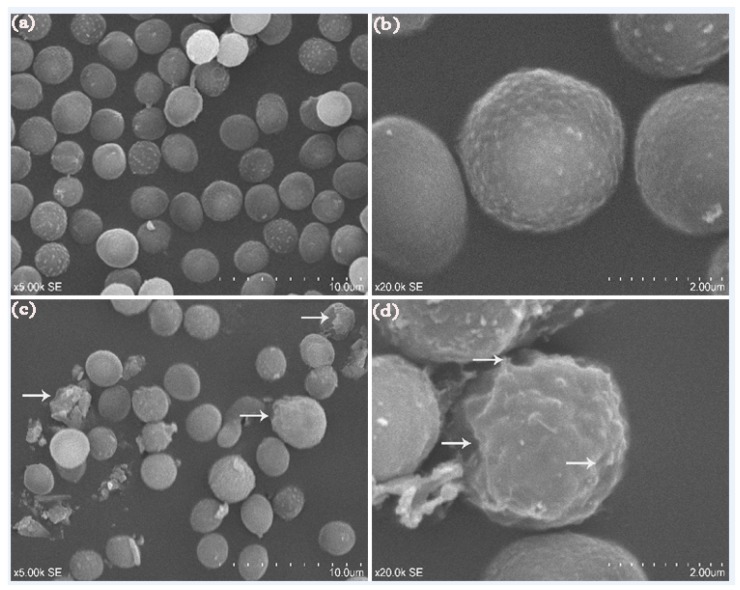
SEM of *P. expansum. P. expansum* conidia treated with distilled water (**a**,**b**), or BanTLP at 60 μM (**c**,**d**) for 12 h. Arrows refer to the morphologic alterations in conidia, such as collapsed cell wall and severe contraction (magnification ×5000, and 20,000).

In our work, other signs of damage to the cell wall of *P. expansum* conidia were found according to analysis with the aid of a scanning electron microscope. After 12 h of incubation at 28 °C, the conidia treated with distilled water displayed a normal and compact morphology (Figure 6a,b), whereas the conidia treated with BanTLP at 60 μM showed irregular modifications. As shown in Figure 6c,d, cell surfaces of numerous conidia in the treatment group appeared to suffer from severe contraction and disruption. 

#### 2.6.2. Transmission Electron Microscope (TEM)

**Figure 7:**
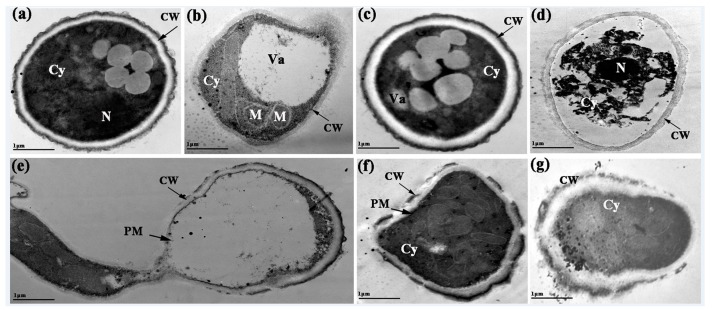
Ultrastructural changes of *P. expansum* observed by TEM. *P. expansum* conidia treated with distilled water (**a**), or BanTLP at 60 μM (**b**–**g**) for 12 h. The scale bar represents 1 μm. Each treatment was repeated three times, and at least two blocks were detected for each replicate. Va, vacuole; CW, cell wall; PM, plasma membrane; Cy, cytoplasm; N, nucleus; M, mitochondria. (Magnification ×30,000).

The effect of BanTLP on the ultrastructural changes of *P. expansum* conidia was estimated using a transmission microscope. After 12 h of exposure treatment, the conidia treated with distilled water showed well-organized cells with a homogeneous cytoplasm, undamaged cell walls, integrated cell membranes, and normal organelles (Figure 7a). In contrast, for the treatment group, the vacuoles of the cells expanded and formed in large numbers, revealing an osmotic stress response in *P. expansum* conidia induced by BanTLP (Figure 7b,c). Additionally, the disorganization of cytoplasm and organelles was evident after BanTLP treatment (Figure 7d,e). A collapsed cell wall can be observed in Figure 7f,g. These ultrastructural changes in conidia may be attributed to enzymatic digestion of the cell wall and increasing membrane permeabilization, followed by the leakage of small molecular substances and ions, and cell metabolism disorder [22].

## 3. Materials and Methods

### 3.1. Fruit and Fungal Materials

Banana (*Musa acuminata*) fruit were transported from Guangzhou to our laboratory at the China Agriculture University immediately after harvest, and stored at 20 °C for complete ripening.

The plant pathogen *P. expansum* was obtained from the microbiology lab of the China Agriculture University. The pathogen strain was cultivated in potato dextrose agar (PDA) at 28 °C for 7 days. Subsequently, the cultures were washed with sterile distilled water containing 0.05% (*v*/*v*) Tween-80. After filtration through four layers of sterile cheesecloth, the spore suspension was obtained in the absence of adherent mycelia. The concentration of spores was determined using a hemocytometer and calibrated to a final concentration at 1 × 10^6^ spores mL^−1^.

### 3.2. Purification and Identification of BanTLP from Banana

Protein extraction was performed according to the method of Xi [28]. In brief, peeled banana (1.5 kg) was homogenized in 180 mL of 1 M Tris (pH 11.2), containing 15% polyvinylpyrrolidone (PVPP). After centrifugation (10,000× *g*, 30 min, 4 °C), the supernatant was collected and dialyzed (MWCO 8000-14000, Serva dialysis tubing) against Tris-HCl buffer (5 mM Tris-HCl, pH 7.5) at 4 °C for 12 h. Subsequently, the crude extract was concentrated five-fold with the aid of a freeze-dryer. The protein content of the crude extract was estimated according to Bradford using bovine serum albumin (Sigma Aldrich, St. Louis, MO, USA) as standard [29].

The BanTLP was purified from the banana extract (300 mL) using a pre-equilibrated anion-exchange column (DEAE–Sephadex A50, 5 × 5 cm) in 30-mM Tris-HCl buffer with pH 8.0, and chromatography was performed in batches. The column was eluted with a gradient of NaCl from 0 to 0.6 M in the same buffer at a flow rate of 1 mL min^−1^. After that, the immunocompetence of every fraction was measured through western blot assay with the rabbit antiserum against BanTLP in banana fruit [30]. Therefore, unbound proteins and the proteins eluted under the low concentration of NaCl from the ion-exchange chromatography (fraction A1) were pooled and collected. Subsequently, the fraction A1 was dialyzed against Tris-HCl buffer (5 mM Tris-HCl, pH 7.5) at 4 °C for 12 h, and then concentrated onto a gel filtration column (70 × 1.6 cm) of Sephadex G50 equilibrated with 30-mM Tris-HCl buffer with pH 8.0, containing 0.1 M NaCl. The column was eluted with the same buffer at a flow rate of 1 mL min^−1^. The eluting peaks (fractions B2 and B3) with the primary antibody reacting bands were pooled and collected, and resolved on a C5 HPLC column (250 × 4.6 mm, 5 μm, Supelco, Bellefonte, PA, USA) equilibrated with 1% trifluoroacetic acid (TFA) in water. Proteins were eluted with a gradient of 0–70% acetonitrile in 0.1% TFA in 40 min. The purified proteins were obtained by collecting protein fraction (C2) with immunocompetence, and dialyzed against Tris-HCl buffer (5 mM Tris-HCl, pH 7.5) at 4 °C for 12 h. The protein after dialysis was concentrated and stored in small aliquots at −20 °C until use.

Sodium dodecyl sulfate-polyacrylamide gel electrophoresis (SDS-PAGE) further confirmed that the purified protein contained only a single polypeptide according to the method described by Laemmli [31], using a miniprotean electrophoresis apparatus (Beijing Liuyi Instrument Factory DYY-151 7C, Beijing, China). The gel with protein bands was stained using a silver-staining technique according to Nesterenko [32].

Protein in-gel digestion assay was conducted according to Liu [11]. Identification of the protein depended on Matrix-assisted laser desorption ionization time-of-flight mass spectrometry (4700 MALDI-TOF/TOF Proteomics Analyzer, Applied Biosystems, Waltham, MA, USA). Mascot data base program (in-house MASCOT server v 2.1, Matrix Science Co., London, UK) was employed to analyze all spectra of proteins against the NCBInr database.

### 3.3. Effect of BanTLP on Spore Germination of P. expansum

Dose-dependent assay was performed by the exposure of spore germination of *P. expansum* to various concentrations of BanTLP following the method described previously [33]. Five concentrations of BanTLP at 0, 5, 10, 15, 20, 40, or 60 μM were prepared in 5 mL of potato dextrose broth (PDB) in 10-mL glass tubes separately. After adding 100 μL of *P. expansum* (1 × 10^6^ spores mL^−1^) to each tube, samples were cultivated at 200 rpm at 28 °C for 12 h and centrifuged (8000× *g*) at 4 °C for 5 min. Afterwards, the conidia were rinsed twice with phosphate-buffered saline (PBS, pH 7.0) and resuspended with the same buffer. Approximately 200 spores per replicate were scored to calculate the germination rate of the test pathogen. The minimal inhibitory concentrations (MICs) of BanTLP against other three common postharvest fungi *Rhizopus stolonifera*, *Botrytis cinerea*, and *Alternaria alternata* were 60, 60, and 30 μM, respectively (Supplementary Materials Table S1). There were three replicates per treatment, and each experiment was repeated twice.

Minimum contact time between BanTLP and spores of *P. expansum* was evaluated following the description by Da with slight modifications [34]. The sterile distilled water (as negative control), 0.05% (*v*/*v*) sodium hypochlorite (as positive control), and BanTLP at 60 μM were prepared in 5 mL of PDB containing *P. expansum* spore suspension (1 × 10^6^ spores mL^−1^) in 10-mL glass tubes. After 0, 2, 4, 6, 8, 10, or 12 h incubation in a shaker of 200 rpm at 28 °C, the conidia were centrifuged (8000× *g*, 5 min, 4 °C), washed twice with 50 mM PBS (pH 7.0) to remove the extra treatment solutions, and resuspended in 5 mL of PDB to a final concentration of 1 × 10^6^ spores mL^−1^. The conidia were incubated in a shaker of 200 rpm at 28 °C for another 12, 10, 8, 6, 4, 2, or 0 h for the germination of spores, respectively. Finally, the germination rate was determined as described above. There were three replicates per treatment, and each experiment was repeated twice.

The pH stability assay was conducted according to Da [34]. The BanTLP at 60 μM were prepared in 5 mL of PDB in 10-mL glass tubes. Cultures were adjusted to different pH values ranging from 2.0 to 14.0 by the addition of 0.05 N HCl or 2 N NaOH. PDB solutions without BanTLP at the same pH series were used as controls. Subsequently, 100 μL of *P. expansum* (1 × 10^6^ spores mL^−1^) were added to each tube and cultivated at 200 rpm at 28 °C for 12 h. The germination rate of *P. expansum* was quantified with the method described above. There were three replicates per treatment, and each experiment was repeated twice.

Thermal stability assay was tested by heating BanTLP dissolved in 20 mM Tris-HCl buffer (pH 7.0) at different temperatures, ranging from 20 to 100 °C for 30 min each in accordance with Theis with slight modifications [35]. With every 10 °C of increasing temperature, the preincubated protein solutions were added to 5 mL of PDB, reaching a final concentration of 60 μM in 10-mL glass tubes. After adding 100 μL of *P. expansum* (1 × 10^6^ spores mL^−1^) to each tube, samples were cultivated at 200 rpm at 28 °C for 12 h. The germination rate of *P. expansum* was determined with the method described above. There were three replicates per treatment, and each experiment was repeated twice.

### 3.4. Effect of BanTLP on the Plasma Membrane of P. expansum

#### 3.4.1. PI Influx

The membrane integrity of *P. expansum* spores was assayed according to Li and Tian with slight modifications [36]. Thus, 100 μL of spore suspensions were transferred to 5 mL of sterile distilled water (as control) or 60 μM BanTLP to obtain a final concentration of 1 × 10^6^ spores mL^−1^. After incubation in a shaker of 200 rpm at 28 °C for 0, 2, 4, 6, 8, 10, or 12 h, the conidia were centrifuged (8000× *g*, 5 min, 4 °C), and washed twice with 50 mM PBS (pH 7.0) to remove the extra mycelia. Subsequently, the spore suspensions were treated with 10 μg mL^−1^ PI (Sigma Aldrich, USA) and incubated in the dark at 30 °C for 5 min. After centrifugation (8000× *g*, 5 min, 4 °C), spores were washed twice with 50 mM PBS (pH 7.0) to remove residual dye, collected, and resuspended in the same buffer for the observation, with the aid of a confocal laser scanning microscopy (Olympus, Fluoview FV1000 Espectral, Tokoy, Japan) equipped with an Olympus digital camera at 546-nm excitation and 590-nm emission wavelengths. The percentage of spores stained with PI was calculated as a proportion of the total spore number, and the number of spores in the bright-field was defined as the total spore number. There were three replicates per treatment, and three fields of view from each microscope slide were randomly chosen and observed.

#### 3.4.2. Potassium Release Assay

Potassium release assay was used to assess the concentration of free potassium ions in suspensions of *P. expansum* spores with the method described previously [37]. The spores exposed to sterile distilled water (as control) or 60 μM BanTLP were incubated in a shaker of 200 rpm at 28 °C for the time intervals described above. After centrifuging at 8000× *g*, for 5 min, the supernatant was collected and used to determine the concentration of extracellular potassium ions with the aid of graphite furnace flame atomic absorption spectroscopy (GFAAS8000, Skyray Instrument, Suzhou, China). The unit of concentration of extracellular free potassium ions was expressed as μg mL^−1^.

#### 3.4.3. Fluorescence Intensities Assay Related to the Membrane Disturbance

Alterations of membrane dynamics were evaluated through fluorescence assay with a DPH probe (Aladdin Industrial Corporation, Los Angeles, CA, USA) according to Yun with slight modifications [38]. Briefly, *P. expansum* spores (1 × 10^6^ mL^−1^) were exposed to sterile distilled water (as control) or 60 μM BanTLP and incubated in a shaker of 200 rpm at 28 °C for 0, 2, 4, 6, 8, 10, or 12 h. Subsequently, the conidia were centrifuged (8000× *g*, 5 min, 4 °C), washed twice with 50 mM PBS (pH 7.0), and then fixed with 0.37% formaldehyde. After freezing with liquid nitrogen and thawing with PBS twice, the samples were incubated with 0.5 mM DPH for 50 min at 30 °C and washed with the same buffer thrice. The conidia were resuspended with PBS and used for the determination of DPH fluorescence intensity with a spectrofluorophotometer (SHIMADZU, RF-5301PC, Kyoto, Japan) at excitation and emission wavelengths of 350 nm and 425 nm, respectively.

#### 3.4.4. ROS Determination

Spores were treated in the same way as Section 3.4.1 described above and incubated in a shaker of 200 rpm at 28 °C for 6 or 12 h. The samples were centrifuged (8000× *g*, 5 min, 4 °C), and washed twice with 50 mM PBS (pH 7.0). Subsequently, the spores of *P. expansum* stained with 10 μM DCFH-DA (Sigma Aldrich, USA) at 37 °C in the dark for 20 min were used to evaluate the generation of ROS in the cell according to Cerioni [39]. After washing twice with PBS, spores were resuspended with the same buffer to a final concentration of 1 × 10^6^ spores mL^−1^. The samples were viewed by fluorescence at 525 nm when excited at 488 nm with confocal laser scanning microscopy. The percentage of positive cells stained by DCFH-DA dye was calculated as a proportion of the total spore number, and the number of spores in bright-field was defined as the total spore number. There were three replicates per treatment, and three fields of view from each microscope slide were observed. 

#### 3.4.5. Determination of TBARS

Spores were treated in the same way as Section 3.4.1 described above and incubated in a shaker of 200 rpm at 28 °C for 0, 2, 4, 6, 8, 10, or 12 h, centrifuged (8000× *g*, 5 min, 4 °C), and then washed twice with 50 mM PBS (pH 7.0). Subsequently, the conidia were subjected to an ultrasound bath thrice for ten minutes each to break the conidia. TBARS were evaluated according to Cerioni [39]. Firstly, 1 mL of conidial extract was mixed with 1 mL of pre-cold 20% trichloroacetic acid (TCA) (*w*/*v*). After centrifugation (19,800× *g*, 5 min, 4 °C), 2 mL of a saturated solution containing thiobarbituric acid (TBA) in 0.1 M HCl and 10 mM of butylated hydroxytoluene were added to the supernatant, which was then incubated in a water bath at 100 °C for 60 min. After cooling at room temperature, the reaction mixture was used to determine the absorbance at 535 nm against a control cuvette in the absence of conidial extract. A molar extinction coefficient of 156 mmol cm^−1^ was employed to calculated the concentration of TBARS. There were three replicates per treatment, and the experiment was performed twice. The resulted was expressed as nmol mg protein.

### 3.5. Effect of BanTLP on Cell Wall Integrity of P. expansum

The integrity of *P. expansum* spores cell walls was evaluated in accordance with Wang, with some modifications [40]. Thus, 100 μL of spore suspensions were transferred into 20 mL of PDB in sterile conical flasks (50 mL) to reach a final concentration of 1 × 10^5^ spores mL^−1^, and cultivated at 200 rpm at 28 °C for 72 h. Subsequently, mycelia were obtained in paper filter (0.2 μm) and resuspended with 5 mL 50 mM PBS (pH 7.0) in a 10-mL sterile glass tube. BanTLP was then added to reach final concentrations of 60 μM. PBS without the antifungal protein served as the control. After incubation at 200 rpm at 28 °C for 6 h or 12 h, samples were collected by centrifugation at 8000× *g* for 10 min at 4 °C, and washed twice with 50 mM PBS (pH 7.0). Subsequently, the mycelia were stained with 50 μg mL^−1^ CFW fluorescent dye (Aladdin Industrial Corporation, USA) in the dark for 15 min. The mycelia were visualized with the aid of confocal laser scanning microscopy at 395-nm excitation and 440-nm emission wavelengths. There were three replicates per treatment, and three fields of view from each microscope slide were observed. 

### 3.6. Morphological and Ultrastructural Changes of P. expansum Conidia

#### 3.6.1. SEM

The sterile distilled water (as control) or BanTLP at 60 μM was prepared in 50 mM PBS (pH 7.0). Subsequently, 100 μL of *P. expansum* spores (1 × 10^6^ spores mL^−1^) were added to each tube and incubated at 200 rpm at 28 °C for 12 h. Then, spores were collected after centrifugation (8000× *g*, 5 min, 4 °C), and washed with 50 mM PBS (pH 7.0) thrice. Then, samples were fixed with 3% glutaraldehyde in 50 mM PBS (pH 7.0) at room temperature for 4 h without agitation and rinsed with PBS four times for 20 min each. After post-fixing in 1% osmic acid at room temperature for 2 h, the samples were washed with double distilled water twice for 15 min. Samples were dehydrated through a graded alcohol series of 30%, 50%, 70%, 80%, 90%, and 95%, then thrice at 100% for 15 min in each series. Following this, samples were incubated in isoamyl acetate overnight. Samples were then subjected to a critical point of dry carbon dioxide and coated with gold. Observation using a scanning electron microscope (S-3400N, SEM system, Hitachi, Tokyo, Japan) was carried out at the College of Biological Science at China Agriculture University.

#### 3.6.2. TEM

Samples were prepared as described for SEM. After fixation with 2% glutaraldehyde at room temperature for 4 h without agitation, the samples were post-fixed in 1% osmic acid at room temperature for 2 h with a gentle shake once and rinsed with PBS twice for 15 min. Afterwards, samples were subjected to dehydration with a graded alcohol series of 30%, 50%, 70%, 80%, 90%, 95%, and 100% (*v*/*v*) for 15 min, respectively, and washed with 100% (*v*/*v*) acetone thrice for 10 min each. After resin infiltration at 35 °C overnight, the ultrathin sections (50–90 nm) were made by using an ultramicrotome with a diamond knife, stained with uranyl acetate solution and lead citrate, and visualized in a transmission electron microscope (H-7500, TEM system, Hitachi, Japan) according to Li [41].

### 3.7. Statistical Analysis

All experiments were performed according to a completely randomized design with three replicates per treatment. The data were subjected to analysis of variance and Duncan’s new multiple range tests to test any significant differences between the means at 5% significance level. Data were evaluated with SPSS Statistics software 19.0 (Chicago, IL, USA).

## 4. Conclusions

In this work, 22.1 -kDa TLPs isolated from banana significantly inhibited postharvest fungus *P. expansum* in a wide pH range (4.0–10.0), and at 20–50 °C. Based on determination of physiological parameters and observations of morphological changes in *P. expansum* exposed to BanTLP, the antifungal protein attacked the cell membranes and induced membrane permeabilization and disturbance. In addition, BanTLP targeted and disrupted cell wall of the test fungus, leading to cell death. Hence, BanTLP acts as a potent antifungal agent with the potential for direct crop protection and the development of pathogen-resistant transgenic plants. However, further research is necessary to elucidate more biological functions of BanTLP in situ in order to highlight its complex mechanisms of host defense resistance against pathogens.

## Figures and Tables

**Table 1:** Protein yields at different stages of purification of thaumatin-like protein from banana (BanTLP).

Fraction	Total Protein from 1.5 kg Flesh (mg)
Crude extract	1038
Unbound fraction from DEAE–Sephadex A50	648
Major peak from Sephadex G50 chromatography	261
After reversed-phase chromatography	58

## Supplementary Materials

The following are available online. The Mascot search results of BanTLP purified from banana, Table S1: The minimal inhibitory concentrations (MIC) (μM) of BanTLP against four common postharvest fungi, Figure S2: The effect of BanTLP at 60 μM on the membrane of *P. expansum* conidia by analyzing PI influx.
