# Supplementary material for: Antifungal Activity of an Abundant Thaumatin-Like Protein from Banana against Penicillium expansum, and Its Possible Mechanisms of Action
Source: Molecules. 2018 Jun 14;23(6):1442. doi: 10.3390/molecules23061442 (PMC6099679; doi:10.3390/molecules23061442)
Supplement: Supplementary file 1 [file molecules-23-01442-s001.pdf]

Supplementary materials

Supplementary Figure. S1. The Mascot search results of BanTLP purified from banana.

Supplementary Table S1. The minimal inhibitory concentrations (MIC) (μM) of BanTLP against four common postharvest fungi.

Supplementary Figure. S2. The effect of BanTLP at 60 μM on the membrane of *P. expansum* conidia by analyzing PI influx.

Mascot Score Histogram

Protein score is -10*Log(P), where P is the probability that the observed match is a random event.
Protein scores greater than 77 are significant (p<0.05).
Protein scores are derived from ions scores as a non-probabilistic basis for ranking protein hits.

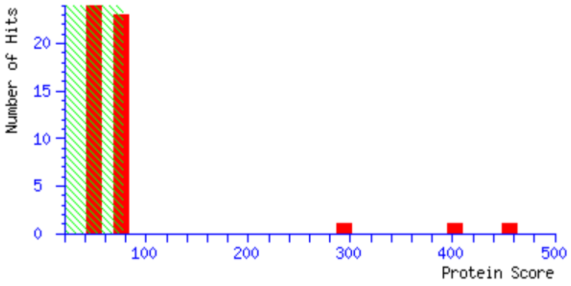

gi|88191901 **Mass:** 22130 **Score:** 414 **Expect:** 1.2e-35 **Matches:** 12
Chain A, Resolution Of The Structure Of The Allergenic And Antifungal Banana Fruit Thaumatin-like Protein At 1.7a

Observed	Mr(expt)	Mr(calc)	ppm	Start	End	Miss	Ions	Peptide
949.5358	948.5285	948.5029	27.0	1 -	8	0	---	-.ATFEIVNR.C
949.5358	948.5285	948.5029	27.0	1 -	8	0	58	-.ATFEIVNR.C
1043.4521	1042.4448	1042.4138	29.7	48 -	57	0	---	R.TGCSFDGSGR.G
1043.4521	1042.4448	1042.4138	29.7	48 -	57	0	32	R.TGCSFDGSGR.G
1370.6466	1369.6393	1369.6085	22.5	169 -	179	1	---	K.RNCPDAYSYPK.D
1421.6731	1420.6658	1420.6228	30.3	135 -	147	0	---	K.APGGCNNPCTVFK.T
1551.7830	1550.7757	1550.7300	29.5	9 -	23	0	---	R.CSYTVWAAAVPGGGR.Q
1551.7830	1550.7757	1550.7300	29.5	9 -	23	0	59	R.CSYTVWAAAVPGGGR.Q
1833.8250	1832.8177	1832.7636	29.5	180 -	195	0	---	K.DDQTTTFTCPGGTNYR.V
2102.0903	2101.0830	2101.0301	25.2	24 -	43	0	---	R.QLNQGQSWTINVNAGTTGGR.I
2102.0903	2101.0830	2101.0301	25.2	24 -	43	0	176	R.QLNQGQSWTINVNAGTTGGR.I
3029.3157	3028.3084	3028.2604	15.9	170 -	195	1	---	R.NCPDAYSYPKDDQTTTFTCPGGTNYR.V

No match to: 804.3040, 986.4307, 1006.5640, 1006.5640, 1057.4685, 1435.6927, 1445.7650, 1458.6826, 1461.7598, 1494.7568, 1534.7555, 1534.7555, 1547.7377, 1550.7223, 1565.8019, 1565.8019, 1583.7809, 1819.8057, 1819.8057, 2056.0181, 2085.0632, 2085.0632, 2117.2581, 2135.0427, 2157.8369, 2171.8599, 2186.8708, 3015.2649, 3015.2649

Supplementary Figure S1. The Mascot search results of BanTLP purified from banana. Ions score is -10*Log(P), where P is the probability that the observed match is a random event. Individual ions scores > 55 indicate identity or extensive homology ($p < 0.05$). Protein scores are derived from ions scores as a non-probabilistic basis for ranking protein hits.

Supplementary Table S1. The minimal inhibitory concentrations (MIC) (μM) of BanTLP against four common postharvest fungi.

BanTLP (μM)	*Penicillium expansum*	*Rhizopus stolonifer*	*Botrytis cinerea*	*Alternaria alternata*
120	-	-	-	-
60	-	-	-	-
30	-	+	+	-
15	+	+	+	+
7.5	+	+	+	+
3.7	+	+	+	+
1.8	+	+	+	+
0.9	+	+	+	+
0.45	+	+	+	+
0.22	+	+	+	+
0.11	+	+	+	+
0.05	+	+	+	+
0.02	+	+	+	+

"-" indicates no fungi detected. "+" indicates fungi detected.

Note: The values of MIC for BanTLP against the common postharvest fungal strains including *P. expansum, R. stolonifera, B. cinerea, and A. alternate* were determined as described by Dananjaya et al. (2017). Briefly, an aliquot of 50 μL fungal spore suspension (1 × 10^6 spores mL^{-1}) was added into 96-well microplates containing 100 μL/well of potato dextrose broth (PDB). Then 100 μL BanTLP was tested in serial dilutions in a concentration range from 0.02 to 120 μM. PDB medium without BanTLP and with sodium hypochlorite were used as negative and positive controls, respectively. Each treatment was carried out in triplicates. All the plates were incubated at 28 ℃ for 48 h and the lowest concentration of BanTLP that did not permit any visible growth after 48 h was considered as the MIC.

Reference cited

Dananjaya, S.H.S.; Udayangani, R.M. C.; Sang, Y.S.; Edussuriya, M.; Nikapitiya, C.; Lee, J.; Zoysa, M.D. In vitro and in vivo antifungal efficacy of plant based lawsone against *Fusarium oxysporum* species complex. *Microbiol. Res.* **2017,** *201*, 21.

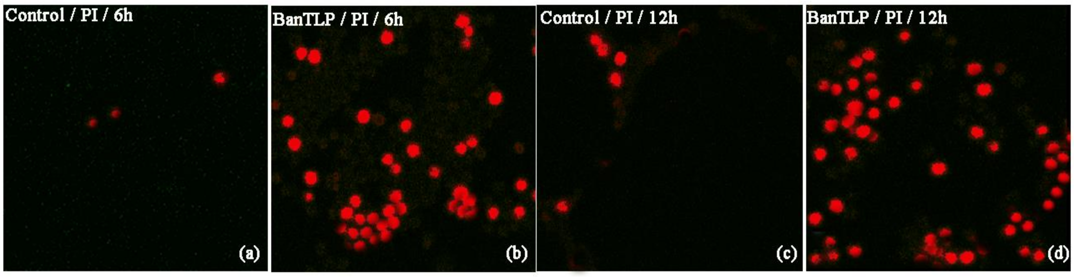

Supplementary Figure S2. Effect of BanTLP at 60 μM on the membrane of *P. expansum* condia by analyzing PI influx. PI fluorescent probe was visualized with the aid of fluorescence microscopy in *P. expansum* conidia treated with distilled water for 6 h (a) and 12 h (c) or treated with BanTLP for 6 h (b) and 12 h (d). There were three replicates per treatment, and three fields of view from each microscope slide were observed.
